# Epidemiology and transmission patterns of *Cryptosporidium* spp., and *Giardia duodenalis* within a One Health framework in rural areas of Eastern Algeria

**DOI:** 10.1017/S0031182024001616

**Published:** 2025-01

**Authors:** Sadiya Maxamhud, Nassiba Reghaissia, AbdElKarim Laatamna, Eleni Gentekaki, Anastasios D. Tsaousis

**Affiliations:** 1 Laboratory of Molecular and Evolutionary Parasitology, RAPID Group, School of Biosciences, University of Kent, Canterbury, Kent, UK; 2 Laboratory of Sciences and Living Techniques, Institute of Agronomic and Veterinary Sciences, University of Souk Ahras, Annaba Road 41000, Souk Ahras, Algeria; 3 Laboratory of Exploration and Valorization of Steppic Ecosystems, Faculty of Nature and Life Sciences, University of Djelfa, Moudjbara Road, BP 3117, Djelfa, Algeria; 4 Department of Veterinary Medicine, University of Nicosia School of Veterinary Medicine, Nicosia, Cyprus

**Keywords:** *Cryptosporidium*, *Giardia duodenalis*, molecular epidemiology, One Health, zoonotic transmission, infectious diseases

## Abstract

Gastrointestinal infections constitute a significant global health concern, particularly in tropical and subtropical regions, caused by various pathogens. Among these, *Cryptosporidium* spp. and *Giardia duodenalis* are noteworthy due to their zoonotic potential. In Algeria, molecular epidemiological data on cryptosporidiosis and giardiasis are limited. To fill this gap, the present study aimed to examine the transmission dynamics of *Cryptosporidium* spp., and *Giardia duodenalis* in various households. A total of 216 samples were collected from the rural Guelma and Souk Ahras provinces, located in the eastern part of Algeria. These included human and animal faeces, as well as water and soil samples. DNA was extracted, followed by nested PCR targeting the *SSU* rRNA gene to detect *Cryptosporidium* spp., while the *gp60* gene was amplified for subtyping. Detection of *G. duodenalis* was performed by qPCR targeting the *SSU* rRNA gene, followed by amplification of *tpi*, *bg* and *gdh* genes for genotyping and subtyping. Several *Cryptosporidium* species, including *C. bovis, C. ryanae, C. andersoni* and *C. parvum*, were identified in human, animal and environmental samples. The zoonotic *C. parvum* subtype IIaA17G2R1 was detected in human, animal and soil samples. *Giardia duodenalis* assemblage B was detected in a human sample, while assemblage E was found in cattle and sheep. The current investigation underscores the importance of the One Health approach in addressing issues related to intestinal parasites, highlighting the need for improved surveillance and control measures in rural settings.

## Graphical abstract

**Figure:**
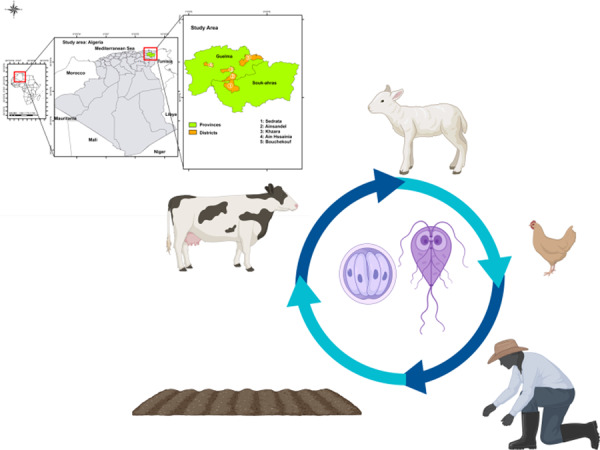

## Introduction

Gastrointestinal infections constitute a common health problem worldwide, with pathogenic species of bacteria, viruses and parasites being the causative agents. Higher rates of these gastrointestinal parasitic infections have been more commonly reported in tropical and sub-tropical areas (Burd and Hinrichs, 2016). *Cryptosporidium* spp. and *Giardia duodenalis* are among the parasites of zoonotic importance. These protozoan pathogens infect the gastrointestinal tract of a broad range of vertebrate hosts, including humans (Ryan *et al.*, 2021). Their corresponding diseases, cryptosporidiosis and giardiasis are among the main causes of diarrhoea in both farm animals and humans (Einarsson *et al.*, 2016; Santin, 2020). Among at least 48 recognized *Cryptosporidium* species, *C. parvum* is a major cause of diarrhoea in calves and lambs, resulting in significant economic losses (Roblin *et al.*, 2023). Moreover, *C. parvum* and *C. hominis* are responsible for most cases of human cryptosporidiosis in both industrialized and non-industrialized countries (Xiao, 2010; Feng *et al.*, 2018). *Giardia duodenalis* is a multispecies complex comprising eight genetically distinct assemblages (genotypes; A-H), of which, A and B are the most reported zoonotic genotypes (Feng and Xiao, 2011; Cacciò *et al.*, 2018; Ryan *et al.*, 2021). Human infections of *Cryptosporidium* and *G. duodenalis* can be asymptomatic or cause mild to severe gastrointestinal signs depending on species/genotypes/subtypes and/or the hosts' immune status.

*Cryptosporidium* spp. and *G. duodenalis* are transmitted via the faecal-oral route, following direct contact with infected hosts/environment or through consumption of contaminated water and foods. Livestock is an important reservoir and source of human infections by shedding of environmentally resilient *Cryptosporidium* oocysts and *Giardia* cysts. Both protozoans have previously been associated with both waterborne and foodborne outbreaks (Ryan *et al.*, 2018; Ryan and Zahedi, 2019; Zahedi and Ryan, 2020). Outbreaks of cryptosporidiosis have been well documented in industrialised countries, including the USA, Ireland, UK, Finland, Germany, Sweden, Norway, South Korea, Australia and Canada (Karanis *et al.*, 2007; Baldursson and Karanis, 2011; Zahedi and Ryan, 2020).Waterborne and foodborne outbreaks of giardiasis have also been reported in North America and Europe (Karanis *et al.*, 2007; Baldursson and Karanis, 2011; Feng and Xiao, 2011). In comparison, data on outbreaks of gastrointestinal infections in non-industrialized countries have been poorly reported, primarily due to the absence of proper surveillance systems.

In Algeria, data on cryptosporidiosis and giardiasis are relatively poorly documented. Studies focusing on the prevalence and/or molecular characterisation of *Cryptosporidium* spp. are fragmentary and include cattle (Akam *et al.*, 2007; Baroudi *et al.*, 2017; Benhouda *et al.*, 2017; Sahraoui *et al.*, 2023), small ruminants (Baroudi *et al.*, 2018; Sahraoui *et al.*, 2019), equids (Laatamna *et al.*, 2013, 2015), dromedary camels (Bouragba *et al.*, 2020; Maxamhud *et al.*, 2023), birds (Baroudi *et al.*, 2013; Laatamna *et al.*, 2017) and fish (Reghaissia *et al.*, 2022). *Cryptosporidium parvum*, *C. hominis* and *C. felis* were recently detected in Algerian HIV patients (Semmani *et al.*, 2023), revealing the presence of *C. parvum* IIa and IId subtype families, specifically IIaA14G2R1, IIaA15G2R1, IIaA16G2R1, IIaA20G1R1, IIaA21G1R1, IIdA16G1 and IIdA19G1 and *C. hominis* Ia and Ib subtype families, specifically IaA14, IaA22R2, IbA13G3 and IbA10G2. *Giardia duodenalis* has previously been detected in cattle (Baroudi *et al.*, 2017), sheep (Sahraoui *et al.*, 2019; Benhassine *et al.*, 2020) and humans (Rebih *et al.*, 2020; Belkessa *et al.*, 2021*a*). Due to insufficient epidemiological data available from Algeria, transmission dynamics of *Cryptosporidium* and *G. duodenalis* among the Algerian populations, specifically those from rural areas, are poorly known.

The close proximity and interactions among humans, livestock and their environments play a significant role in disease transmission (Squire and Ryan, 2017). Livestock breeding is the main agricultural activity in the rural areas, particularly sheep breeding in the steppes and cattle and poultry farming in the high plateaus of Algeria. The absence of improved husbandry and management practices increases the risk for zoonotic transmission of both parasites. As such, understanding the transmission dynamics under a comprehensive framework requires a One Health approach.

The present study investigates the transmission dynamics of *Cryptosporidium* spp. and *G. duodenalis* between humans and animals from different households, and their environment in rural areas in Northeastern Algeria.

## Materials and methods

### Ethics statement

The ethics committee of the Laboratory of Exploration and Valorization of Steppic Ecosystems from the Faculty of Nature and Life Sciences, Ziane Achour University-Djelfa, Algeria, approved the collection of human samples in this study (001 EVES/FSNV/2021). The study protocol was explained to each family guardian (head of the family), and informed consent for collecting faecal samples was obtained.

### Study area and sample collection

**Figure 1.**
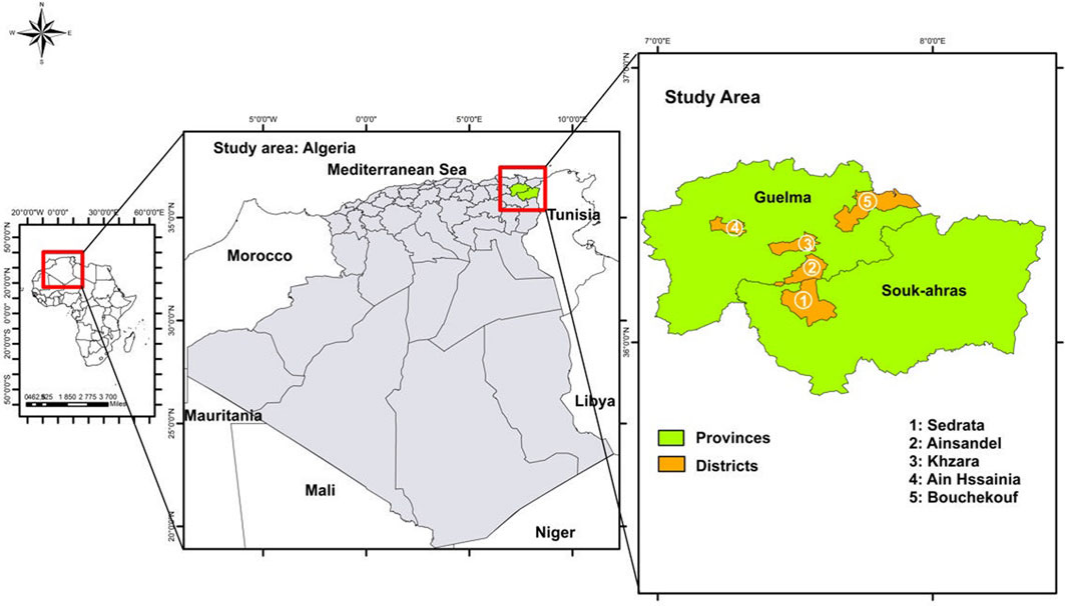
Map of Algeria, showing the 5 sampling regions in the 2 provinces: Guelma and Souk Ahras.

The present study was carried out between May and October 2022 in 5 rural areas; 4 in Guelma province and 1 in Souk Ahras province, both located in Northeastern Algeria (Fig. 1). Guelma is a highland region located in the Northeastern part of Algeria. It is characterized by diversified plant cover and sub-humid climate in the central and north parts, while in the south, the climate is semi-arid. In this region, there are valleys and dams, including Oued Seybouse. Souk Ahras is also a high plateau area located in the north-east and its climate is mainly semi-arid. This province has a dense hydrographic network, formed by a multitude of permanent and temporary watercourses such as Oued Medjerda, Oued Mellag and Oued Charef. In both provinces, livestock husbandry consists mainly of cattle, sheep and goat breeding. A total of 39 different households were included in this study, including 26 from Guelma and 13 from Souk Ahras. Each household was composed of 3–11 individuals, including both children and adults, at the time of sampling. These families lived in close proximity to various domestic animal species, including ruminants, birds, equids, dogs and cats. Moreover, stray dogs as well as wildlife, such as carnivores and boars are known to circulate in these areas. In both Guelma and Souk Ahras, households have access to nearby natural water sources.

The inclusion criteria for participating in the study were living in rural areas and being clinically healthy (no gastrointestinal signs as well as no signs of any other disease). In this study, a total of 216 samples were collected. These consisted of human stools (*n* = 65), animal faeces (*n* = 135), water samples (*n* = 4) and soil samples (*n* = 12). More precisely, the collected animal samples consisted of cattle (*n* = 67), sheep (*n* = 56), chickens (*n* = 6), turkeys (*n* = 2), goats (*n* = 2), cat (*n* = 1) and horse (*n* = 1). Sterile tubes were distributed to members of the included households for stool collection. None of the sampled individuals showed any signs of diarrhoea at the time of sampling. The individuals sampled from each household ranged from 15% to 100% of the total residents within each household. In some households, no human samples, as well as no animal samples, were collected, only environmental. Data for sampled individuals, including the date of sampling, age, gender and their livestock, were obtained from the family guardian of each household. Faeces of animals were collected directly from the rectum or from the ground immediately after defecation. All faecal samples were preserved in DNA/RNA shield RNA solution at 4°C until molecular analysis.

Soil samples were collected close to the houses of the included families (*n* = 8) or, in some cases, from the nearest soil habitat to houses (*n* = 4). Water samples (*n* = 4) were sourced from drinking water, including 2 samples from natural water sources, one sample from a well and another from tap water.

### DNA extraction and molecular analysis

The genomic DNA was extracted from 200 mg of both stool and soil samples using a PureLink™ Microbiome DNA Purification Kit (Invitrogen, Carlsbad, California, USA) according to the manufacturer's protocol. DNA from water samples was extracted using E.Z.N.A. Water DNA Kit (OMEGA, Stamford, CT, USA) according to the manufacturer's protocol. The extracted DNA was stored at −20°C until PCR analysis.

Detection of *Cryptosporidium* was performed using a nested PCR approach amplifying partial sequences of the small subunit rRNA (SSU) and 60-kDa glycoprotein (gp60) genes as previously described (Pinto *et al.*, 2021; Reghaissia *et al.*, 2022).

The process of *Cryptosporidium* spp. detection initially involved targeting and amplifying the SSU rRNA gene sequence for species/genotype determination. The primary reaction employed the primers CRY_SSU_F1 (5′-GATTAAGCCATGCATGTCTAA-3′) and CRY_SSU_R1 (5′-TTCCATGCTGGAGTATTCAAG-3′), resulting in a product size of 723 bp. Subsequently, for the second reaction, the forward primer CRY_SSU_F2 (5′-CAGTTATAGTTTACTTGATAATC-3′) and the reverse primer CRY_SSU_R2 (5′-CCTGCTTTAAGCACTCTAATTTTC-3′) was used, with an expected product size of approximately 631 bp (Lindergard *et al.*, 2003). The primary PCR mixture, with a volume of 25 μL, contained 1 μL of template gDNA, 0.4 μm of each primer and 12.5 μL of 2 × PCRBIO Taq Mix Red. Amplification was carried out with an initial denaturation at 94°C for 2 min, followed by 24 cycles of denaturation at 94°C for 50 s, annealing at 53°C for 50 s and extension at 72°C for 1 min, concluding with a final extension step at 72°C for 10 min. For the secondary PCR reaction, 1 μL of the primary PCR product was utilized as a template, and the remaining mixture was prepared similarly to the primary reaction. In the second PCR condition, an initial denaturation was conducted at 94°C for 2 min, followed by 30 cycles of denaturation at 94°C for 50 s, annealing at 56°C for 30 s, and extension at 72°C for 1 min, concluding with a final extension step at 72°C for 10 min.

Furthermore, the amplification of the gp60 gene for *Cryptosporidium parvun* species subtyping was conducted via nested PCR using specific primers. In the primary reaction, the primers AL3531 (5′-ATAGTCTCCGCTGTATTC-3′) and AL3535 (5′-GGAAGGAACGATGTATCT-3′) were employed, and in the secondary reaction, the primers AL3532 (5′-TCCGCTGTATTCTCAGCC-3′) and AL3534 (5′-GCAGAGGAACCAGCATC-3′) were used (Pinto *et al.*, 2021). Both primary and secondary PCR mixtures contained 2 μL of gDNA or the primary PCR product, 0.2 μm of each primer and 15 μL of 2 × PCRBIO Taq Mix Red in a total volume of 30 μL. PCR conditions included an initial denaturation at 94°C for 3 min, followed by 35 cycles of denaturation at 94°C for 45 s, annealing at 50°C for 45 s and extension at 72°C for 1 min, with a final extension step at 72°C for 7 min. The same PCR conditions were applied to both primary and secondary reactions.

DNA of positive samples was subsequently extracted and purified using the GeneJET Gel Extraction Kit (ThermoFisher Scientific, CA, USA).

Detection of *G. duodenalis* was performed by a probe-based quantitative PCR (qPCR) targeting a partial sequence (62 bp) of the SSU gene as previously described (Verweij *et al.*, 2004; Hove *et al.*, 2007; Maxamhud *et al.*, 2023). Each reaction was composed of a 20 μL total volume, comprising 10 μL of 1× Luna Universal Probe qPCR Master Mix (New England Biolabs, Massachusetts, USA), 0.8 μL of 0.4 μM *G. duodenalis*-specific primers; *Giardia*-80F (5′-GACGGCTCAGGACAACGGTT-3′) and *Giardia*-127R (5́-TTGCCAGCGGTGTCCG-3′), 0.4 μL of 0.2 μm *G. duodenalis*-specific probe (FAM-5′-CCCGCGGCGGTCCCTGCTAG-3′-black hole quencher), 1 μL of 0.5 μg μL^−1^ bovine serum Albumin (BSA) (Promega Madison, WI, USA), 5 μL of nuclease-free H_2_O (Promega Madison, WI, USA) and 2 μL of extracted DNA. Amplification was conducted with QuantStudio™ three with PCR conditions as follows: an initial denaturation step at 95°C for 2 min, followed by 50 cycles of 95°C for 15 s, annealing at 57°C for 30 s and a final elongation step at 72°C for 30 s.

The determination of *G. duodenalis* assemblages was performed using nested PCRs targeting the *triosephosphate isomerase* (*tpi*), *β-giardin (bg)* and the *glutamate dehydrogenase (gdh)* genes as previously described (Cacciò *et al.*, 2002, 2008; Sulaiman *et al.*, 2003). DNA of positive samples was subsequently extracted and purified using the GeneJET Gel Extraction Kit (ThermoFisher Scientific, CA, USA).

### Sequencing, DNA cloning and phylogenetic analyses

Sequencing of PCR products from both *Cryptosporidium* spp. and *G. duodenalis* positive samples was performed bidirectionally at Eurofins laboratory (Cologne, Germany). Positive samples for either *Cryptosporidium* spp. or *G. duodenalis* that exhibited further evidence of mixed infections or non-specific binding/amplification were cloned as described by Betts *et al.* (2018).

Obtained sequences were visualized and edited manually with the software SnapGene Viewer v.6.0.2 and ChromasPro v.2.1.10. Subsequently, the identity of obtained sequences was checked using BLAST search (www.ncbi.nlm.nih.gov/blast). Moreover, two distinct datasets were constructed, 1 for SSU rRNA gene sequences for *Cryptosporidium* spp. and another for only *tpi* gene sequences of *G. duodenalis*. Sequence analyses for *bg* and *gdh* genes were not included due to the poor quality of the obtained sequences. These sequence datasets were aligned using MAFFT v.7 (Katoh and Toh, 2010). Automated removal of ambiguous regions from sequence alignment was performed using trimAL v.1.4 (Capella-Gutiérrez *et al.*, 2009). Alignments consisted of 31 taxa (sequences) and 467 sites for *G. duodenalis* and 71 taxa and 1674 for *Cryptosporidium.* Maximum likelihood (ML) phylogenetic trees with bootstrap support were inferred by using IQTREE v.2.0.3 (Nguyen *et al.*, 2015) and MEGA7 v.7.0 (Kumar *et al.*, 2016), and subsequently visualized using FigTree 1.4.4 (http://tree.bio.ed.ac.uk/software/figtree/). Furthermore, to assess the genetic diversity among multiple sequences generated in the present study, the genetic distance was calculated using Kimura2 parameter with gamma distribution as implemented in MEGA7 (Kumar *et al.*, 2016). This analysis determined the percentage of genetic variation between the sequences, enabling simultaneous comparisons across multiple sequences. Generated nucleotide sequences from the present study were deposited in the GenBank database under the following accession numbers: PP484686, PP484689, PP484691- PP484697, PP484699, PP484701 and PP484705 for *C. bovis*, PP484687 and PP484702-PP484704 for *C. parvum*, PP481965, PP481967- PP481968 and PP481972- PP481973 for *C. parvum* IIaA17G2R1, PP481966 for *C. parvum* IIaA16G2R1, PP481969 for *C. parvum* IIaA13G1R1, PP481970 for *C. parvum* IIA15G2R1, PP481971 for *C. parvum* IIdA16G1, PP481974 for *C. parvum* IIaA14G1R1, PP484685, PP484688, PP484698 and PP484700 for *C. andersoni*, PP484690 for *C. ryanae* and PP481975-PP481979, PP481981-PP481986 for *G. duodenalis* assemblage E and PP481980 for *G. duodenalis* assemblage B.

## Results

The ages of the individuals involved in this study varied. Children aged 4–14 years (*n* = 23), adolescents aged 15–17 years (*n* = 3) and adults aged 22 to 48 years (*n* = 31) were included. Eight individuals had no age data recorded. Moreover, the number of females and males was 17 and 40, respectively. The remaining 8 human samples that were included had no gender data recorded.

At least one protozoan was identified in 62% (24/39) of households investigated, involving either animals, humans or both. *Cryptosporidium* spp. was detected in 41% (16/39) of the households, while *G. duodenalis* was detected in 56% (22/39) of the households included in this study. Both *Cryptosporidium* spp. and *G. duodenalis* were detected in 33% (13/39) of the households included (Table 1).

**Table 1.** Distribution of *Cryptosporidium* species and subtypes and *Giardia intestinalis* assemblages in animals, humans and environmental samples from examined households

Province	Rural locality	Code of household no.	No. of human examined samples/No. of positive cases	No. of animal examined samples/No. of positive cases	Water sample (+ or −)	Soil sample (+ or −)
**Guelma**	Ain Sandel	**01**	(0/0) (no human sampling)	Cattle (1/1) (1 × *C. bovis*) Sheep (2/0) /////////////	No sampling	No sampling
	Ain Sandel	**02**	(0/0) (no human sampling)	Cattle (2/1) (1 × *C. bovis*, 1 × *G. intestinalis)*^a^^,^^b^ Sheep (1/0) //////////////	No sampling	No sampling
	Ain Sandel	**03**	(0/0) (no human sampling)	Cattle (2/2) (2 × *C. parvum* IIaA17G2R1) Sheep (6/3) (1 × *C. bovis*, 1 × *G. intestinalis*)^a^^,b^ (2 × *G. intestinalis*)^b^	No sampling	No sampling
	Ain Sandel	**04**	(0/0) (no human sampling)	Cattle (1/0) ////////////// Sheep (4/2) (1 × *C.bovis*, 1 × *G. intestinalis*)^a^^,b^, (1 × *C. parvum* IIaA15G2R1)	No sampling	No sampling
	Ain Sandel	**05**	(1/0)	Sheep (5/0) (1 × *G. intestinalis*)^b^ Goat (1/1) (1 × *C. parvum* IIdA16G1b, 1 × *C. parvum* IIaA14G1R1)^a^	No sampling	No sampling
	Ain Sandel	**06**	(6/3) (3 × *G. intestinalis*)^b^	Cattle (3/2) (1 × *C. parvum* IIaA17G2R1, 1 × *C. bovis*)^a^, (1 × *G. intestinalis*)^b^ Sheep (3/1*)* (1 × *C. parvum* IIaA13G1R1, 1 × *G. intestinalis*)^a^^,b^	No sampling	Negative
	Ain Sandel	**07**	(1/1) (*G. intestinalis* B)	Cattle (3/1) (1 × *C. ryanae*)	No sampling	Negative
	Ain Sandel	**08**	(1/1) (*C. bovis*)	Cattle (2/0) /////////////// Sheep (2/2) (2 × *C.bovis*) Chicken (1/0) /////////// Cats (1/0) /////////////	No sampling	Negative
	Ain Sandel	**09**	(5/2) (2 × *G. intestinalis*)^b^	Cattle (4/2) (1 × *C. bovis*, 1 × *G. intestinalis* E)^a^, (1 × *G. intestinalis* E) Goat (1/1) (1 × *G. intestinalis*)^b^ Chicken (1/0) //////////////	Negative	No sampling
	Ain Sandel	**10**	(1/0)	Cattle (5/1) (1 × *G. intestinalis* E) Sheep (19/13) (4 × *C. bovis*, 4 × *G. intestinalis* E)^a^^,^^c^ *(2* × *G. intestinalis* E*)* (2 × *C. bovis*, 2 × *G. intestinalis*)^a^^,b^, (1 × *C parvum*, 1 × *G. intestinalis*)^a^^,b^, (7 × *G. intestinalis*)^b^	No sampling	Negative
	Bouchegouf	**11**	(6/0)	Cattle (3/0) /////////// Sheep (2/2) (1 × *G. intestinalis* E), (1 × *G. intestinalis*)^b^	No sampling	No sampling
	Ain Hssainia	**12**	(3/3) (3 × *G. intestinalis*)^b^	Chicken (2/2) (1 × *C. andersoni*, 1 × *G. intestinalis*)^a^^,b^, (1 × *G. intestinalis*)^b^ Turkey (2/2) (1 × *C. parvum*, 1 × *C. andersoni*)^a^, (1 × *G. intestinalis*)^b^ Sheep (3/3) (3 × *G. intestinalis*)^b^ Cattle (7/6) (1 × *C. parvum*, 1 × *G. intestinalis*)^a^^,b^, (5 × *G. intestinalis*)^b^	No sampling	Negative
	Khzara	**23**	1/0	Chicken (1/0) /////////////	No sampling	Negative
	Khzara	**24**	4/0	0/0 (no animal sampling)	(*G. intestinalis*)^b^	No sampling
	Khzara	**25**	1/0	Sheep (1/0)	No sampling	No sampling
	Ain Sandel	**26**	5/1(1 × *G. intestinalis*)^b^	0/0 (no animal sampling)	No sampling	(*G. intestinalis*)^b^
	Ain Sandel	**27**	0/0 (no human sampling)	Sheep (1/0) //////////////////	No sampling	No sampling
	Ain Sandel	**28**	6/0	0/0 (no animal sampling)	Negative	No sampling
	Ain Sandel	**29**	1/0	0/0 (no animal sampling)	No sampling	Negative
	Ain Sandel	**30**	0/0 (no human sampling)	Cattle (2/0) /////////////// Sheep (1/0) ////////////////	No sampling	No sampling
	Ain Sandel	**31**	(0/0) (no human sampling)	Sheep (2/0) ///////////////	No sampling	No sampling
	Ain Sandel	**32**	(0/0) (no human sampling)	Sheep (4/1) (1 × *G. intestinalis*)^b^ Cattle (1/0) ////////////////	No sampling	No sampling
	Ain Sandel	**33**	(1/0)	Cattle (2/0) ///////////////	No sampling	No sampling
	Ain Sandel	**34**	0/0 (no human sampling)	Cattle (1/0) ///////////////	No sampling	No sampling
	Ain Sandel	**35**	0/0 (no human sampling)	Cattle (1/0) ///////////////	No sampling	No sampling
	Ain Sandel	**36**	0/0 (no human sampling)	0/0 (no animal sampling)	No sampling	Negative
**Souk-Ahras**	Sedrata	**13**	(0/0) (no human sampling)	Cattle (4/4) (1 × *C. andersoni*), (3 × *G. intestinalis*)^b^	No sampling	(*G. intestinalis*)^b^
	Sedrata	**14**	(1/0)	Cattle (5/4) (1 × *C. andersoni*, 1 × *G. intestinalis)*^a^^,^^b^, (3 × *G. intestinalis*)^b^ Chicken (1/0) ///////////////// Horse (1/0) ///////////////////	No sampling	*C. parvum* IIaA17G2R1
	Sedrata	**15**	(0/0) (no human sampling)	Cattle (5/5) (1 × *C. parvum* IIaA16G2R1, 1 × *G. intestinalis*)^a^^,b^, (4 × *G. intestinalis*)^b^	No sampling	No sampling
	Sedrata	**16**	(3/1) (1 × *C. parvum* IIaA17G2R1, 1 × *G.intestinalis*)^a^^,b^	(0/0) (no animal sampling)	No sampling	No sampling
	Sedrata	**17**	(0/0) (no human sampling)	Cattle (3/2) (1 × *C. bovis*, 1 × *G. intestinalis*)^a^^,b^, (1 × *G. intestinalis*)^b^	No sampling	No sampling
	Sedrata	**18**	5/0	0/0 (no animal sampling)	No sampling	Negative
	Sedrata	**19**	(0/0) (no human sampling)	Cattle (5/4) (4 × *G. intestinalis*)^b^	No sampling	No sampling
	Sedrata	**20**	3/1 (1 × *G. intestinalis*)^b^	(0/0) (no animal sampling)	No sampling	No sampling
	Sedrata	**21**	3/1 (1 × *G. intestinalis*)^b^	(0/0) (no animal sampling)	No sampling	No sampling
	Sedrata	**22**	3/1 (1 × *G. intestinalis*)^b^	(0/0) (no animal sampling)	No sampling	No sampling
	Unknown	**37**	5/0	(0/0) (no animal sampling)	(*G. intestinalis*)^b^	No sampling
	Unknown	**38**	0/0 (no human sampling)	Cattle (1/0)	No sampling	No sampling
	Unknown	**39**	(0/0) (no human sampling)	Cattle (4/1) (1 × *G. intestinalis*)^b^	No sampling	No sampling

a Mixed infection.

b *G. intestinalis* positive samples with unsuccessful genotyping.

c Three different sheep samples showing mixed infection with *C. bovis*, and *G. intestinalis* assemblage E.

In total, out of the 135 animal samples screened, *Cryptosporidium* spp. was detected in 19% (26/135), which included 18% (12/67) of the sampled cattle, 20% (11/56) of the sampled sheep, 50% (1/2) of the sampled goats, 17% (1/6) of the sampled chickens and 50% (1/2) of the sampled turkeys. More precisely, *C. bovis* was present in 11 animals [42% of cattle (5/12) and 55% of sheep (6/11)], *C. parvum* in 10 animals [42% of cattle (5/12), 27% (3/11) of sheep, one goat and one turkey], *C. andersoni* in four animals [17% (2/12) of cattle, one chicken and one turkey] and *C. ryanae* (*n* = 1) in one cattle. One cattle from household N°6 and one turkey from household N°12 exhibited a mixed infection with *C. parvum*/*C. bovis* and *C. parvum*/*C. andersoni*, respectively. Samples that tested positive for *C. parvum* were subtyped as IIaA17G2R1 (*n* = 3) and IIaA16G2R1 (*n* = 1) in four different cattle, and IIaA15G2R1 (*n* = 1) and IIaA13G1R1 (*n* = 1) in two different sheep. In addition, one goat exhibited a mixed infection with two different *C. parvum* subtypes, namely IIdA16G1b and IIaA14G1R1. Three *C. parvum* samples, specifically one from sheep, one from turkey and one from cattle, could not be subtyped due to unsuccessful amplification of the *gp60* gene. The sampled cat and horse were negative for both *Cryptosporidium* spp. and *G. duodenalis*.

Out of the 65 human samples examined in this study, 2 were positive for *Cryptosporidium* spp. This included one testing positive for *C. bovis* and the other for *C. parvum* (subtype IIaA17G2R1). These two samples were collected from two separate households. Neither participant showed signs of diarrhoea at the time of sampling. Moreover, in the household N° 8 (Table 1), *C. bovis* was detected in both humans and sheep.

**Figure 2.**
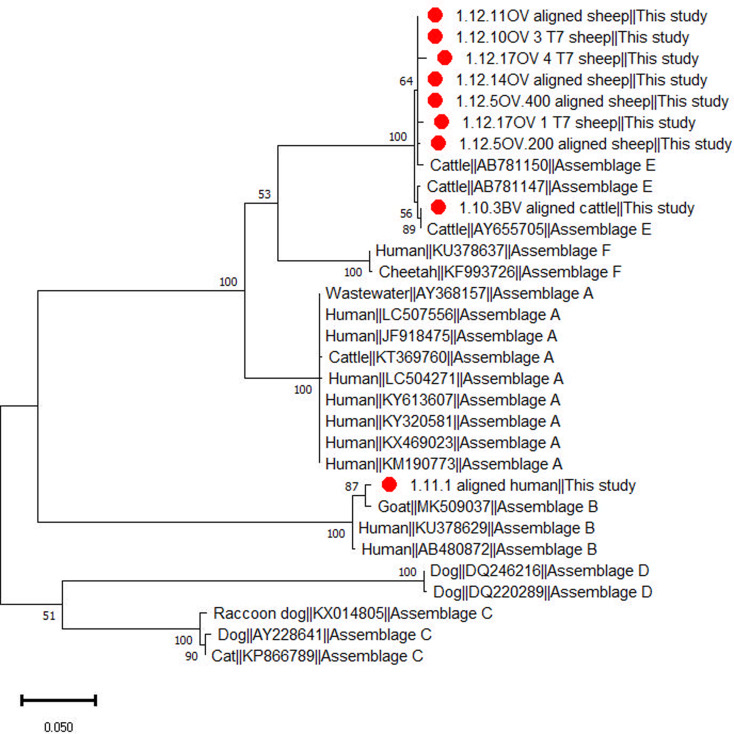
Phylogenetic relationship among *Giardia duodenalis* assemblages inferred based on partial sequences of the triosephosphate isomerase (*tpi*) gene using the maximum likelihood method.

*Giardia duodenalis* was detected by qPCR in 44% (59/135) of the animal samples, consisting of 45% (30/67) of cattle, 45% (25/56) of sheep, 50% (1/2) of goats, 33% (2/6) of chickens and 50% (1/2) of turkeys. Based on BLAST and phylogenetic analysis of *tpi* gene sequences (Fig. 2) ten animal samples, including seven from sheep and three from cattle, were successfully genotyped to assemblage E. In total, 17 animals exhibited mixed infections involving *Cryptosporidium* spp. and *G. duodenalis*. This included three cattle and eight sheep infected with *C. bovis* and *G. duodenalis*, two cattle and two sheep infected with *C. parvum* and *G. duodenalis* and one cattle and one chicken each infected with *C. andersoni* and *G. duodenalis*. Notably, among the sheep infected with *C. bovis* and *G. duodenalis*, four *G. duodenalis* samples were genotyped as assemblage E. These results were further confirmed with phylogenetic analyses (Fig. 2; Supplementary Fig. 1)

*Giardia duodenalis* was detected in 14 samples of human origin, of which only 1 was successfully genotyped as assemblage B. One human sample exhibited a mixed infection with *C. parvum* (IIaA17G2R1) and *G. duodenalis*. Six of the *G. duodenalis* infected humans were children aged between 4 and 11 years, while 8 were adults aged between 27 and 84 years.

*Giardia duodenalis* was also detected in humans and animals residing within the same household. This included three different households, namely the households N° 6, 9 and 12 from Guelma province (Table 1). Additionally, *G. duodenalis* was identified in both humans and soil samples within the household N° 26 in Guelma.

Among the four analysed water samples, two natural sources were positive for *G. duodenalis* and have been successfully genotyped. These water-positive samples originated from two different households (N° 24, 37), and no human samples tested positive for *G. duodenalis*. Among the 12 analysed soil samples, 1 was positive for *C. parvum* (subtype IIaA17G2R1) and two for *G. duodenalis* which could not be successfully genotyped. One of the *G. duodenalis* positive soil samples was traced back to the household N°26 where a human sample also tested to be positive for *G. duodenalis*. The other *G. duodenalis* positive soil sample originated from the household N° 13 where three cattle were also positive to *G. duodenalis*. The soil sample that tested positive for *C. parvum* (subtype IIaA17G2R1) originated from a household, namely household N° 14, where no humans nor animals tested positive for this *C. parvum* subtype.

The samples that tested positive for *G. duodenalis* and were successfully genotyped originated from four distinct households, all located in Ain Sandel within the Guelma region. Among these, six sheep tested positive for assemblage E, exhibiting identical sequences, all belonging to the same household (N°10). Furthermore, two additional cattle samples, from a different household (N°9), also tested positive for assemblage E and shared identical sequences.

The sequence of *C. andersoni* was identical in 2 samples, 1 from a turkey and the other from a cattle. These samples originated from households N°12 and 14 in Ain Hssainia, Guelma province and Sedrata, Souk Ahras province, respectively (Table 1).

Additionally, a total of seven samples were positive for *C. bovis*, comprising 4 sheep, 2 cattle, and 1 human sample, shared identical sequences. These samples were collected from five distinct households (households N°1, 2, 4, 8 and 10), with three sheep originating from the household N°10. All these households were situated in Ain Sandel within the Guelma region. Furthermore, two other samples, both from cattle, also tested positive for *C. bovis* and exhibited identical sequences with each other but not with any other positive samples for *C. bovis*. One sample was obtained from the household N°17 in Ain Sandel, Guelma and the other from the household N°6 in Sedrata, Souk Ahras.

## Discussion

This study took place in five rural areas, where traditional livestock breeding is the main activity. People in these areas live in close contact with their livestock (such as ruminants) as well as carnivorous pets (such as cats). Moreover, people rely on a local and traditional diet where cows' milk and meat are essential food sources. Hence, these communities are more exposed to the risk of transmission of infectious diseases. Due to these characteristics, this particular community was selected for this One Health study to gain insight into the transmission dynamics of 2 zoonotic protozoans: *Cryptosporidium* and *G. duodenalis*.

Among the animal species raised in the investigated households, cattle, sheep, goats, chickens and turkeys were infected with either *Cryptosporidium* or *G. duodenalis*, or both. This study reported the presence of *G. duodenalis* in domestic birds (chickens and turkeys), which could comprise a reservoir that contributes to environmental contamination in rural areas. In humans from the examined households, *Cryptosporidium* spp. and *G. duodenalis* were detected 3% and 21%, respectively. In the Algerians, human infections of both parasites have rarely been explored. A low prevalence (0.2%) of *Cryptosporidium* spp. was reported by Belkessa *et al.*, (2021*b*), while another study reported an infection rate of 9.5% in HIV patients (Semmani *et al.* 2023). Infection rates of *G. duodenalis* ranged of 0.3–67% (Benouis *et al.*, 2013; Hamaidi-Chergui *et al.*, 2013; Rebih *et al.*, 2020; Belkessa *et al.*, 2021*a*, 2021*b*). Various drivers can influence these prevalence variations such as sample size, detection methods, characteristics of persons involved in the study, and the study design.

Four *Cryptosporidium* species were detected in livestock raised in the investigated households, including *C. andersoni*, *C. bovis*, *C. ryanae and C. parvum*. *Cryptosporidium bovis* was the most detected species in cattle and sheep in the present study. In Algeria, *C. bovis* has been previously reported in pre-weaned calves (Baroudi *et al.*, 2017; Benhouda *et al.*, 2017; Ouakli *et al.*, 2018; Sahraoui *et al.*, 2023) and in dromedary camels (Maxamhud *et al.*, 2023). This species is well adapted to cattle, particularly in postweaning calves (Santin, 2020), but it has been sporadically detected in other livestock, such as sheep (Ryan *et al.*, 2005; Squire and Ryan, 2017). In the present study, one asymptomatic individual tested positive for *C. bovis* along with a sheep from the same household, suggesting possible direct transmission between the two. Human infections by *C. bovis*, specifically in cattle farm workers, have been reported in few cases, and therefore, its zoonotic importance seems to be minor (Khan *et al.*, 2010; Ng *et al.*, 2012; Helmy *et al.*, 2013). *C. parvum* was the second most detected species in livestock, including cattle, sheep, goats and turkey. *C. parvum* infects a broad host range and is the most common zoonotic *Cryptosporidium* species worldwide. The human sample that tested positive for *C. parvum* IIaA17G2R1, originated from a household where no animal sampling was done. Six different *C. parvum* subtypes that belonged to two zoonotic subtype families IIa and IId were identified in the present study. The subtype IIaA17G2R1 was identified in both cattle and humans with IIaA16G2R1 also detected in cattle. *Cryptosporidium* subtypes IIaA15G2R1 and IIaA13G1R1 were identified in sheep. In addition, one goat showed a mixed infection with IIaA14G1R1 and IIdA16G1b. In farm animals worldwide, subtype family IIa is commonly found in cattle, while subtype family IId is more commonly found in sheep and goats. However, subtype family IIa has also been reported in small ruminants (Xiao and Feng, 2017; Feng *et al.*, 2018). Cattle can contribute to the transmission of *C. parvum* subtypes of family IIa to sheep and goats (Feng *et al.*, 2018). Even though the infected human, who tested positive for *C. parvum* IIaA17G2R1, originated from a household where no animal and environmental samples were collected, it is important to note that cattle remain an important source of transmission for humans. In Algeria, the IIaA17G2R1 subtype has previously been detected in dromedary camels (Maxamhud *et al.*, 2023) and fish (species *Sparus aurata*) (Reghaissia *et al.*, 2022). The present study reports, for the first time, the occurrence of IIaA17G2R1 in humans from Algeria. Moreover, the IIaA16G2R1 subtype has previously been detected in calves (Baroudi *et al.*, 2017; Ouakli *et al.*, 2018; Sahraoui *et al.*, 2023), fish (Reghaissia *et al.*, 2022) as well as in HIV patients (Semmani *et al.*, 2023). The subtype IIaA15G2R1 that was identified in sheep in this study has also previously been reported in Algeria in calves (Sahraoui *et al.*, 2023) and dromedary camels (Maxamhud *et al.*, 2023) as well as in HIV patients (Semmani *et al.*, 2023). The subtype IIaA15G2R1 is a common (mainly in cattle) and a hypertransmissible zoonotic subtype reported worldwide (Feng *et al.*, 2018) and herein we suggest that sheep could be an important source for human transmission and subsequent infection. The *Cryptosporidium parvum* subtype IIaA13G1R1 identified in sheep has not previously been reported in Algeria. Among the two examined goats, one exhibited a mixed infection with IIdA16G1b and IIaA14G1R1. Our results represented the detection of subtype families IIa and IId in goats from Algeria. The subtype IIaA14G1R1 has not been reported in Algeria in cattle or other livestock such as small ruminants. Other previous Algerian studies reported the occurrence of IIdA16G1 in lambs and calves (Sahraoui *et al.*, 2019, 2023) and in HIV patients (Semmani *et al.*, 2023).

In the present study, *C. andersoni* was detected in cattle, chicken and turkey. This gastric species is predominant in juvenile and adult cattle, whereas it has been found less frequently in other livestock (Deng *et al.*, 2017; El-Alfy *et al.*, 2019; Santin, 2020). In Algeria, *C. andersoni* has been previously reported in calves (Baroudi *et al.*, 2017). Mixed farming and close contact among cattle, sheep and domestic birds in the investigated rural areas could have contributed to the infection of non-specific hosts such as chickens and turkeys. *Cryptosporidium andersoni* is not considered a major zoonotic species, but it has been reported in humans in some countries (Santin, 2020; Ryan *et al.*, 2021). Additionally, *C. ryanae* was identified in one sample of cattle origin. This species is predominant in postweaned calves, but it has been found in small ruminants (Santin, 2020). In Algeria, *C. ryanae* has previously been reported in preweaned calves (Benhouda *et al.*, 2017; Ouakli *et al.*, 2018). *Cryptosporidium ryanae* has no zoonotic impact and, to the best of our knowledge, has not been reported in humans (Ryan *et al.*, 2021).

In the examined animals from the present study, positive *G. duodenalis* samples were successfully genotyped in cattle and sheep and identified as assemblage E. In Algeria, assemblage E has been reported from one study in lambs (Benhassine *et al.*, 2020). This assemblage is common in hoofed animals such as ruminants and pigs (Feng and Xiao, 2011; Ryan and Zahedi, 2019). Only one sample was successfully identified as assemblage B in the examined humans. Assemblage B has been previously reported in Algerian children and adults (Belkessa *et al.*, 2021*a*) but is commonly reported in humans (Ryan and Zahedi, 2019), in contrast to assemblage E (Fantinatti *et al.*, 2016; Zahedi *et al.*, 2017). Different households showed the presence of *G. duodenalis* in humans and their livestock; however, despite our unsuccessful attempts to genotype the *Giardia* samples, this study emphasizes the possibility of infected livestock contributing to human infection and environmental contamination.

Among the four water samples screened for the presence of both parasites, twp natural water sources showed contamination by *G. duodenalis*. These contaminated samples originated from two different households, and the screened humans were negative for *G. duodenalis* and *Cryptosporidium* spp. Although livestock living close to these drinking water sources were not sampled, their contamination was likely associated with the presence of nearby infected animals.

Soil samples showed one positive for *C. parvum* (subtype IIaA17G2R1) and two for *G. duodenalis*. The soil sample tested positive for *C. parvum* IIaA17G2R1 and was collected in an area close to a household where cattle were infected with *C. andersoni*. However, as shown in the other examined households, cattle can act as an important reservoir and source for the environmental spread of this subtype. In the case of *G. duodenalis*, environmental soil contamination can be associated with faeces from infected livestock. It is important to note that the environmental contamination by *Cryptosporidium* oocysts and *Giardia* cysts can effectively contribute to human and livestock infections through environmental transmission. Waterborne outbreaks caused by *Cryptosporidium* spp. and *G. duodenalis* have been reported mainly in developed countries, while there is a lack of data on these outbreaks in developing countries (Karanis *et al.*, 2007; Baldursson and Karanis, 2011). The present study provides preliminary data on the occurrence of *C. parvum* (subtype IIaA17G2R1) in soil and *G. duodenalis* in both soil and drinking water in Algeria. Epidemiological data on cryptosporidiosis and giardiasis waterborne outbreaks are poorly documented in developing countries, including those in the African continent. This is primarily due to a lack of technological and surveillance resources such as reliable diagnostic methods and effective reporting systems (Karanis *et al.*, 2007; Baldursson and Karanis, 2011).

This study has some limitations, particularly concerning the imbalance of sampling and limited genotype/subtype-specific data, which did not allow for a clear understanding of the transmission dynamics. However, the role livestock and environment play in the possible transmission of human infections of *Cryptosporidium* spp. and *G. duodenalis* was demonstrated here. This study underscored the importance of adopting a One Health approach, particularly in rural settings where these parasites pose a significant challenge to human and animal health. This study did not only shed light on the genetic diversity of *Cryptosporidium* spp. across various hosts in Algeria, but also contributed to our growing knowledge of the molecular epidemiology of cryptosporidiosis and giardiasis in the country. Future comprehensive investigations are necessary in rural areas to enhance the understanding of transmission dynamics of these organisms. By emphasizing the relevance of the One Health concept as well as the transmission dynamics of these intestinal parasites between humans, animals and their shared environment, future effective control measures could be established.

## Supporting information

Maxamhud et al. supplementary material Maxamhud et al. supplementary material

## Data Availability

Generated nucleotide sequences from the present study were deposited in the GenBank database under the following accession numbers: PP484686, PP484689, PP484691-PP484697, PP484699, PP484701 and PP484705 for *C. bovis*, PP484687 and PP484702-PP484704 for *C. parvum*, PP481965, PP481967PP481968 and PP481972-PP481973 for *C. parvum* IIaA17G2R1, PP481966 for *C. parvum* IIaA16G2R1, PP481969 for *C. parvum* IIaA13G1R1, PP481970 for *C. parvum* IIA15G2R1, PP481971 for *C. parvum* IIdA16G1, PP481974 for *C. parvum* IIaA14G1R1, PP484685, PP484688, PP484698 and PP484700 for *C. andersoni*, PP484690 for *C. ryanae* and PP481975-PP481979, PP481981-PP481986 for *G. duodenalis* assemblage E and PP481980 for *G. duodenalis* assemblage B.

## Supplementary material

The supplementary material for this article can be found at https://doi.org/10.1017/S0031182024001616
