# Supplementary figures and images for: Epidemiology and transmission patterns of Cryptosporidium spp., and Giardia duodenalis within a One Health framework in rural areas of Eastern Algeria
Source: Parasitology. 2024 Dec 18;152(1):51–60. doi: 10.1017/S0031182024001616 (PMC12088920; doi:10.1017/S0031182024001616)

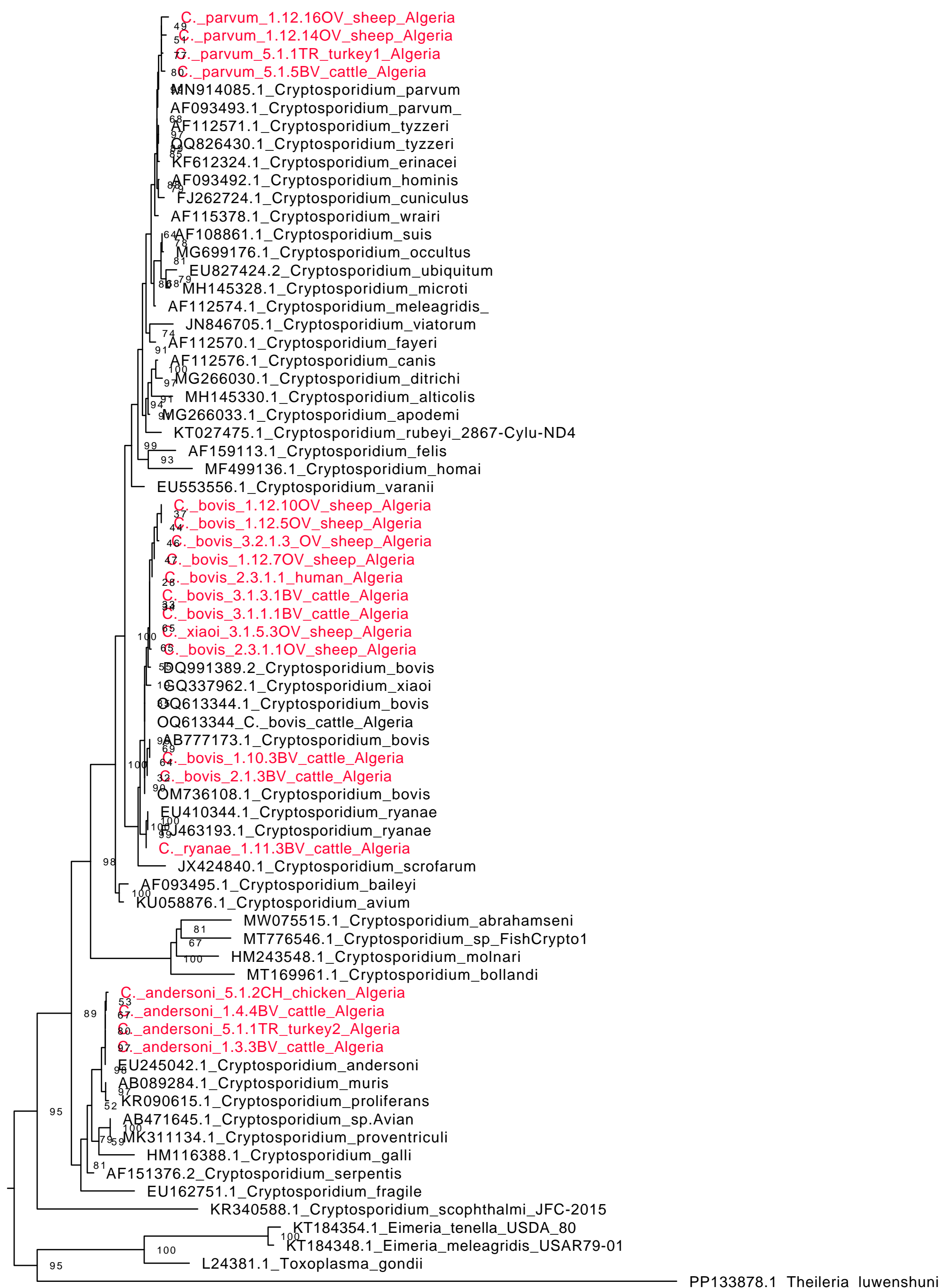

0.09

Supplement: Maxamhud et al. supplementary material [file S0031182024001616sup001.pdf]
